# Iris pigment epithelial cysts in a newborn

**DOI:** 10.3205/oc000044

**Published:** 2016-04-22

**Authors:** Shabnam Zargar, Kevin John Prendiville, Eladio Martinez

**Affiliations:** 1 University of California, Riverside School of Medicine, UCR Health Family Medicine Center, Palm Springs, USA; 2 Shaaf Eye Center, Rancho Mirage, USA; 3 Instituto Mexicano del Seguro Social, Hospital General Regional no. 1, IMSS Hospital General De Zona N0 6 Tecate, Tecate, Mexico

**Keywords:** iris pigment epithelial cysts, amblyopia, newborn

## Abstract

**Purpose:** We report a case of iris pigment epithelial cysts in a newborn and discuss the importance of an accurate diagnosis for prevention of amblyopia.

**Methods:** We describe a case of an abnormal red reflex seen on a newborn exam.

**Results:** A full-term female born via normal spontaneous vaginal delivery without any complications was seen in the newborn nursery. She was noted to have an abnormal eye exam. Pupils were large with circular dark excrescences of the iris pigment epithelium. She was referred to a pediatric ophthalmologist where she was noted to fixate and follow faces. No afferent pupillary defect was seen. OD red reflex was normal whereas OS red reflex was blocked mostly by dark excrescences. A 2–3 mm dark brown lesion was seen in the OD iris and a 3–5 mm dark brown lesion was seen in the OS iris, consistent with a pupillary iris pigment epithelial cyst. Central visual axis was clear OU. Glaucoma was not present and patching was not performed. Observations and clinical photographs were recommended with follow-up in three months.

**Conclusion:** Iris pigment epithelial cysts are uncommonly seen in children. The primary care provider first seeing a newborn must be aware of lesions obscuring a red reflex with appropriate follow-up. Follow-up in three months with IOP measurements is recommended. Iris pigment epithelial cysts in children may be a cause of amblyopia, thus prompt evaluation is important for prognostic purposes and the prevention of amblyopia.

## Introduction

Iris pigment epithelial cysts are uncommonly seen in children. These cysts are benign lesions of the anterior segment [1]. Iris pigment epithelial cysts may be classified as primary or secondary, with further classifications of primary epithelial cysts as peripheral, midzonal, pupillary, or cysts of the iris stroma [2]. The majority of primary cysts of the iris pigment epithelium are benign, located at the iridociliary junction, and are round or oval with thin walls and a fluid filled center [2], [3]. Many of the primary iris pigment epithelial cysts remain undetected or undiagnosed until later in childhood or young adulthood with a careful slit lamp exam [1]. We report a case of primary pupillary iris pigment epithelial cysts, noted in a newborn female upon gross ophthalmologic exam. 

## Case description

**Figure 1:**
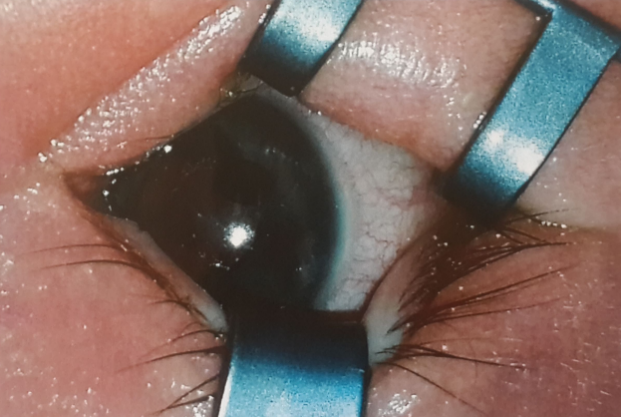
Photo OD with superior eyelid in the inferior position on the screen

**Figure 2:**
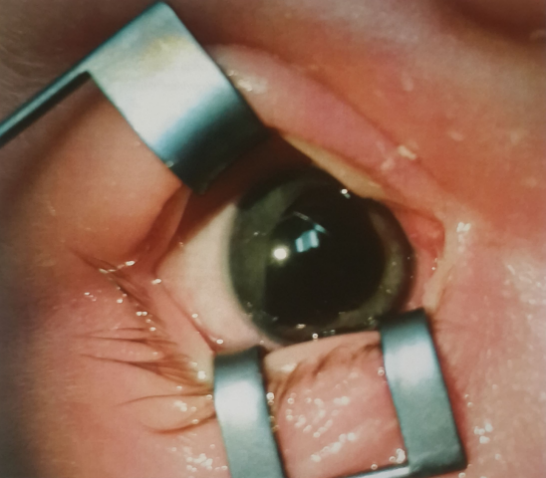
Photo OS with superior eyelid in the inferior position on the screen

A full-term female infant was born via normal spontaneous vaginal delivery without any complications. Prenatal labs were normal and no amniocentesis was performed. Prenatal medications included Prozac and prenatal vitamins. No family history of eye anomalies were reported. On the first day of life, the patient was noted to have an abnormal red reflex. Pupils appeared large and poorly reactive with circular dark excrescences of the iris pigment epithelium. Patient was referred to a pediatric ophthalmologist, where she was seen at 3 weeks of life. Examination was performed while awake. She was noted to fixate and follow faces. OD pupil was 6–7 mm, slowly reactive, OS pupil 8–9 mm, slowly reactive, and there was no afferent pupillary defect. OD red reflex was normal, whereas OS red reflex was noted to be mostly blocked by dark excrescences. IOP was 12 bilaterally, measured by Reichert tonopen applanation. Eyelids, conjunctiva, and cornea were noted to be normal bilaterally. Anterior chambers were noted to be clear and deep, aside from temporal shallowing in OS. Upon examination of the OD iris, a 2–3 mm dark brown lesion, uniform in color without mottling or orange color pigment was noted (Figure 1 (Fig. 1)). This was out of the central visual axis with minimal displacement of the iris. The iris in OS revealed a 3–5 mm dark brown lesion also with uniform color and without mottling or orange color pigment (Figure 2 (Fig. 2)). Temporal displacement of the overlying anterior part of the iris was noted, with a clear central visual axis. Lenses OU were clear without displacement or subluxation. OU motility ductions were full. OU refraction was +2.00 sph. OU retina noted to have normal macula and vessels. Optic nerves OU were sharp with C/D 0.1. It was concluded that this newborn patient had a lesion involving the iris OU, with the OS lesion larger than OD, with features of a pupillary iris pigment epithelial cyst. Central visual axis was clear OU, with the left cyst covering over 1/3 of the pupil and the right cyst not in the pupil significantly. Ultrasound biomicroscopy was not performed. Our patient was thus diagnosed with a peripheral iris pigment epithelial cyst of the OD and a pupillary iris pigment epithelial cyst of the OS. The parents were reassured that the lesions appeared to be non-amblyogenic. She is to be observed by pediatric ophthalmology with clinical photographs every three months initially and then every six months.

## Discussion

Iris epithelial cysts are uncommon in children. These benign cysts tend to be bilateral and multiple and affect females more commonly than males [1], [3]. The etiology of primary anterior iris cysts in unknown, although theories include a separation of the epithelial layers at the junction between the pigmented iris and non-pigmented ciliary body [1]. Most iris cysts of childhood are primary pigment epithelial cysts, requiring no treatment, whereas an iris stromal cyst is generally aggressive and requires aspiration or surgical excision [2]. Most anterior segment anomalies are diagnosed with a thorough history and ophthalmologic exam. Pupillary and peripheral iris pigment epithelial cysts may be seen in infancy. The importance of recognizing iris pigment epithelial cysts in a newborn lies in amblyopia prevention. Amblyopia is the most common cause of visual impairment in childhood and affects 2–3 children out of 100 [4]. As amblyopia is preventable, it becomes imperative to recognize possible amblyogenic lesions as soon as possible. Recent studies have found several risk factors associated with amblyopia [5]. This has significant repercussions in pediatrics, as amblyopia can become permanent if not treated in childhood. Pupillary lesions may also be mistaken for other anomalies, such as coloboma. Therefore, prompt ophthalmologic exam and accurate diagnosis is necessary for prognostic purposes along with frequent follow-up and tonometry measurements.

Uncommonly, large lesions may produce focal angle-closure [3]. Iris pigment epithelial cysts may be observed over time, with clinical photographs to monitor change in the cysts. In our patient, the lesions appeared benign and non-amblyogenic. In conclusion, the importance of recognizing iris pigment epithelial cysts in a pediatric patient cannot be underestimated as a means of observation and prevention of amblyopia. 

## Notes

### Patient consent

The legal guardians of the patient have consented to the submission of the manuscript to the journal.

### Competing interests

All authors certify that they have no affiliations with or involvement in any organization or entity with any financial interest (such as honoraria; educational grants; participation in speakers’ bureaus; membership, employment, consultancies, stock ownership, or other equity interest; and expert testimony or patent-licensing arrangements), or non-financial interest (such as personal or professional relationships, affiliations, knowledge or beliefs) in the subject matter or materials discussed in this manuscript.
